# Comparative study of peritoneal adhesions after intraperitoneal implantation in rats of meshes of polypropylene *versus* polypropylene/polyglecaprone *versus* polyester/porcine collagen^1^

**DOI:** 10.1590/s0102-865020190060000003

**Published:** 2019-08-19

**Authors:** Waston Gonçalves Ribeiro, Diego Vinnicyus Santos Rodrigues, Francisco Felipe Moreira Atta, Izabelle Smith Frazão Ramos, Fabiola Nassar Sousa Frazão, Orlando Jorge Martins Torres, Marcos Bettini Pitombo

**Affiliations:** I Master, Fellow PhD degree, Postgraduate Program in Health Sciences, Faculty of Medical Sciences, Universidade do Estado do Rio de Janeiro (UERJ), Brazil. Conception and design of the study, technical procedures, analysis and interpretation of data, statistics analysis, manuscript writing.; II Resident, General Surgery Residency Program, Hospital Universitário, Universidade Federal do Maranhão (HU-UFMA), Sao Luis-MA, Brazil. Technical procedures, acquisition of data.; III Graduate student, School of Medicine, UFMA, Sao Luis-Ma, Brazil. Technical procedures, acquisition of data.; IV PhD, Chairman, Full Professor, Department of Surgery, UFMA, Sao Luis-Ma, Brazil. Conception and design of the study, critical revision.; V PhD, Associate Professor, Department of General Surgery, Faculty of Medical Sciences, UERJ, Rio de Janeiro-RJ, Brazil. Conception and design of the study, interpretation and analysis of data, critical revision, final approval.

**Keywords:** Hernia, Ventral, Surgical Mesh, Tissue Adhesions, Rats

## Abstract

**Purpose:**

To Compare the extent and intensity of adhesions formed between the intra-abdominal organs and the intraperitoneal implants of polypropylene mesh *versus* polypropylene/polyglecaprone *versus* polyester/porcine collagen used for correction of abdominal wall defect in rats.

**Methods:**

After the defect in the abdominal wall, thirty Wistar rats were placed in three groups (ten animals each) for intraperitoneal mesh implant: polypropylene group, polypropylene/polyglecaprone group, and polyester/porcine collagen group. The macroscopic evaluation of the extent and intensity of adhesions was performed 21 days after the implant.

**Results:**

The polypropylene group had a higher statistically significant impairment due to visceral adhesions (p value = 0.002) and a higher degree of intense adherence in relation to polypropylene/polyglecaprone and polyester/porcine collagen groups (p value<0.001). The polyester/porcine collagen group showed more intense adhesions than the polypropylene/polyglecaprone group (p value=0.035).

**Conclusions:**

The intraperitoneal implantation of polypropylene meshes to correct defects of the abdominal wall caused the appearance of extensive and firm adhesions to intra-abdominal structures. The use of polypropylene/polyglecaprone or polyester/porcine collagen tissue-separating meshes reduces the number and degree of adhesions formed.

## Introduction

The incisional abdominal hernia can be defined as a hernia protrusion that develops in the topography of a previous surgical incision or a traumatic injury to the abdominal wall. It is one of the most frequent complications after elective or emergency abdominal surgeries. The incidence is 10-20%, reaching a higher rate (30-40%) in patients with associated risk factors^1-2^. In the United States of America, approximately four to five million abdominal surgeries are performed annually, resulting in an approximate incidence of 500.000 new cases of incisional hernias^1-3^. About 365.000 incisional hernioplasties were performed in the US in 2006 at a cost of $ 3.2 billion^4^. Due to the increase in the survival of patients with traumatic and infectious abdominal catastrophes, the number of large incisional hernias has increased, as well as the complexity of their surgical management^1,2-5^. The repair of large abdominal wall hernias is technically challenging. It is associated with a long hospitalization, difficulties and complications in the healing process, intra-abdominal hypertension, high rate of re-operations, readmissions and hernia recurrences, with a consequent increase in overall costs of treatment^1,6-7^.

The use of meshes for the repair of abdominal wall hernias is a widely discussed concept, and is performed by conventional or videolaparoscopic tension-free techniques. The development and the clinical use of polypropylene meshes in hernia repair of the abdominal wall is considered a historical milestone in the treatment of hernias^8^. The prostheses used in abdominal hernioplasties may be biological or synthetic. The synthetic meshes are composed mostly of polypropylene or polyester, or expanded polytetrafluoroethylene (PTFEe), or polyvinylidene fluoride (PVDF). In turn, biological meshes (bioprostheses) are composed of bovine or porcine pericardium (xenogenic) and a human acellular dermal matrix (allogeneic)^9-10^. The use of synthetic meshes for the repair of abdominal wall hernias has become important because it is able to reduce failure rates and recurrence of hernia after surgical treatment, solidifying as a gold standard in the management of abdominal hernias^1,6-11^.

Several biological, chemical and physical characteristics of meshes, synthetic and bioprosthetic, differentiate the prostheses used for the repair of hernia defects of the abdominal wall. Ideally, such prosthetic implants should be chemically inert, have an inflammatory and fibroplastic response that leads to the incorporation of the mesh, but not result in an intense foreign body-type reaction to the extent that it compromises the elasticity of the abdominal wall or limits its movement. It should also be biocompatible, strong, resistant to infection, nonimmunogenic or carcinogenic, minimally bioreactive, affordable, moldable, sterilizable, and easy to handle^9-12^. The availability of a variety of different types of meshes for the surgical treatment of abdominal hernias leads to an inevitable conclusion: the ideal mesh is not yet available. However, meshes made of polypropylene or polyester are the most commonly used for this purpose^11^.

Although the use of meshes for the repair of abdominal wall hernia defects is widely accepted and widespread, and has a positive impact in reducing failure rates and relapses^6^, the intra-abdominal use of most available prostheses is restricted. After implantation intraperitoneally, they can determine important adhesions between the surface of the mesh and the intra-abdominal viscera, especially with the small intestine, the colon and the epiploon, favoring the appearance of chronic abdominal pain, intestinal obstruction, enterocutaneous fistulas, chronic infection of the mesh, and a consequent need for surgeries to treat such complications, or even complicating conventional or laparotomic and videolaparotomic approaches after hernioplasty, leading to a significant morbidity and additional costs^13-15^.

The videolaparoscopic treatment of incisional hernias is usually performed using intraperitoneally implanted meshes. Therefore, they come in direct contact with the abdominal viscera. In this scenario, the meshes are called tissue separators. They are a coated, composite, double face, bilayer, or anti-adherence barrier^2-9^. Tissue separating meshes present a reticulated parietal face that, in contact with the underlying muscle-aponeurotic and peritoneal plane, favors the fibroplasia process and the incorporation of the mesh, providing an adequate tensile strength to the tissue. It also has a visceral, laminar face that, in contact with the viscera, can enhance the process of mesothelialization (formation of the neoperitoneum), thus reducing the risk of developing adhesions and their deleterious effects^13-16^. Tissue separating meshes can be divided into two groups: meshes with a permanent barrier, in which the visceral face is not degraded or absorbed, and meshes with absorbable (temporary) barriers, in which the visceral surface has a transitory effect^11-17^. Usually, the components of these barriers determine a wide variety of inflammatory responses and fibroplasia, resulting in a diverse rate of adhesions between the mesh and intra-abdominal viscera. This suggests that experimental and clinical studies are needed to achieve a definitive clinical efficacy of tissue separating meshes.

A comparative experimental study was carried out on three different types of synthetic prostheses commonly used for the repair of incisional hernias. A low weight polypropylene mesh was used with no non-stick barrier and two tissue-separating meshes, a light polypropylene composite associated with polyglecaprone and the other a polyester composite with porcine collagen. All meshes were implanted intraperitoneally aiming an analysis of the extent and intensity of adhesions formed between the mesh and the intra-abdominal anatomical structures determined by the three different types of prostheses.

## Methods

The study respected the Brazilian legislation on the use of experimental animals (Lei Arouca no. 11.794/2008) and the standards of the Brazilian College of Animal Experimentation (COBEA). It was analyzed and approved by the Ethics Committee on the Use of Animals (CEUA) of Universidade Federal do Maranhão, registration no. 23115.011726/2016-51.

Surgical procedures were performed at the Experimental Surgery Research Laboratory, Hospital Universitário, UFMA. Thirty Wistar rats (*Rattus norvegicus albinus*), adult males, with a mean weight of 307 ± 33 g, and 60 days of life, were selected from the *Bioterium* of UFMA. The animals were kept in a polypropylene cage under constant environmental conditions, receiving a ration for rats and water ad libitum for seven days for adaptation. There was noise control. The temperature was 22°C ± 2°C, the relative humidity was 40% to 60%, and the light/dark cycles were of 12/12 hours.

**Figure 1:**
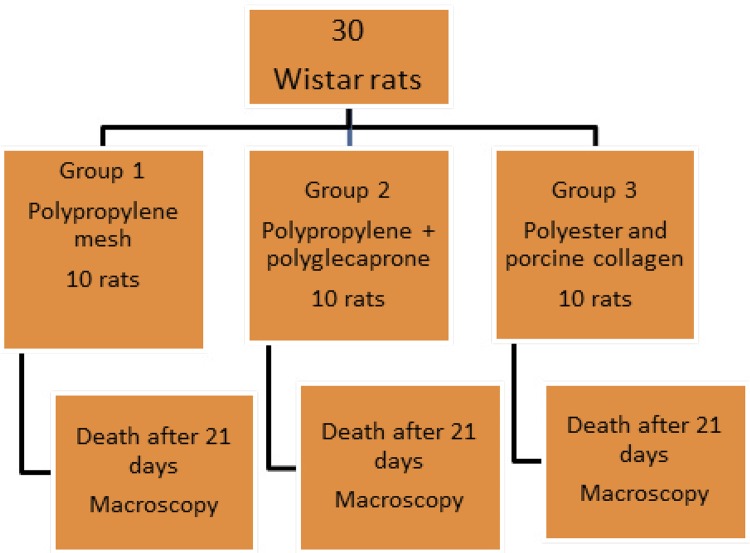
Design of the experimental research comparing the intraperitoneal implant of three different types of meshes (polypropylene *versus* polypropylene with polyglecaprone *versus* polyester with porcine collagen) in Wistar rats.

Rats were randomly assigned into three groups of ten animals (Fig. 1). A median laparotomy and the repair of a repaired abdominal wall defect were performed with a 4x3 cm mesh implanted intraperitoneally according to the selected group. Group 1: polypropylene mesh - OPTILENE® Mesh (B.Braun Surgical SA, Barcelona, Spain); group 2: polypropylene mesh with polyglecaprone - PHYSIOMESH® Flexible Composite Mesh (Ethicon, Somerville, NJ, USA); and group 3: a polyester mesh with collagen previously hydrated with 0.9% saline for one minute - SYMBOTEX® Composite Mesh (Covidien, Trévoux, France).

After a fasting of 12 hours, the rats were anesthetized with a mixture of 2% xylazine hydrochloride at a dose of 10 mg/kg and 10% ketamine hydrochloride at a dose of 100 mg/kg intramuscularly using a hypodermic needle of 13 mm x 4.5 mm on the posterior border of the left thigh. The evaluation of the efficacy of anesthesia was verified by the loss of the corneal-eyelid reflex and the reflex of the interdigital pressure. A manual epilation of the abdominal region was performed. The antisepsis was performed with polyvinyl-pyrrolidone-iodine, and then a fenestrated field was placed.

**Figure 2:**
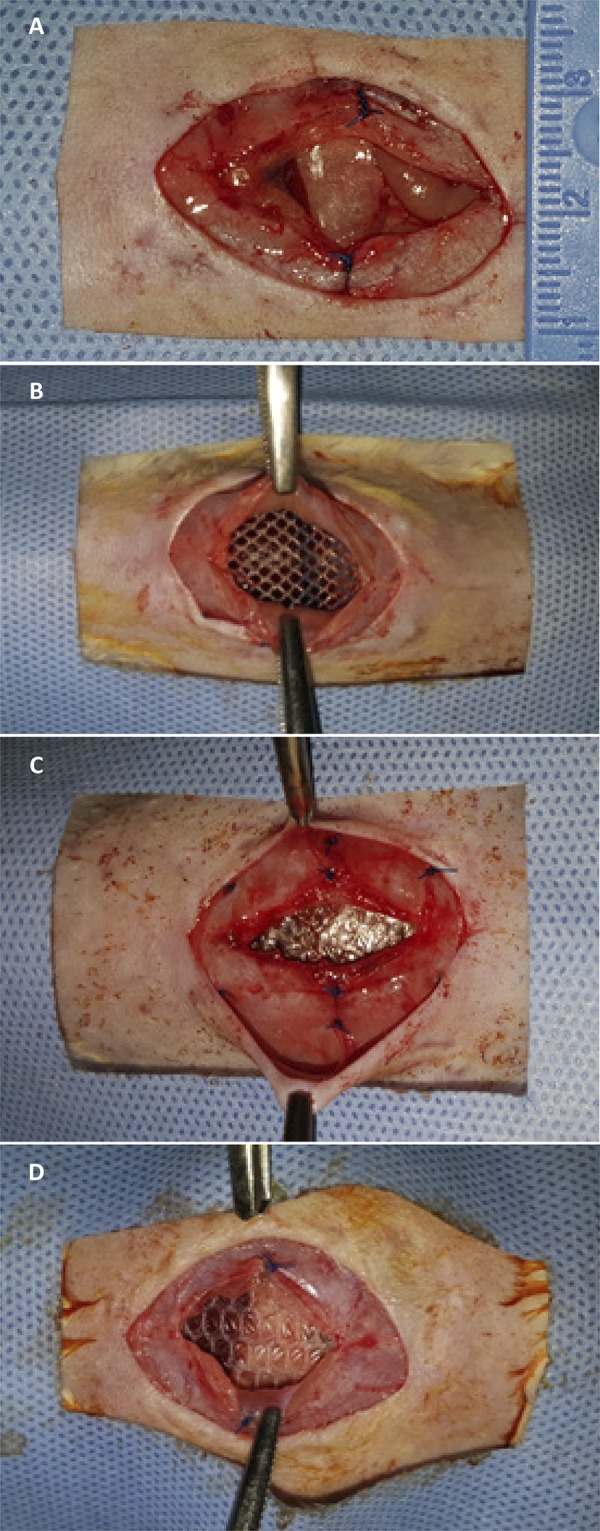
Preparation of the defect in the abdominal wall (A) and fixation of the polypropylene mesh (B), polypropylene with polyglecaprone (C) and polyester with porcine collagen (D).

The animals were submitted to a laparotomy through a medial incision with 4 cm of extension immediately caudal to the xiphoid appendix, and dieresis planes with dissection between the cutaneo-adipose and musculoaponeurotic planes up to 2 cm on each side of the median line followed by opening of the abdominal cavity in the alba line measuring 2.5 cm with one suture point with polypropylene 4.0 (PROLENE® Ethicon, Somerville, NJ, USA) on each side of the incision, everting the edges of the abdomen rectum muscle, without covering the peritoneum, thus creating a defect with 2.5 x 1.5 cm (area = 3.75 cm^2^), without any need for abdominal wall resection^12^ (Fig. 2). According to the allocation, one of the synthetic meshes with 4 x 3 cm (area = 12 cm^2^) was implanted intraperitoneally by six transfixing “U” points in the musculoaponeurotic plane with a polypropylene 4.0 thread (PROLENE®, Ethicon, Somerville, NJ, USA) applied at the four corners of the mesh and at the midpoint between the caudal and cranial point on each side thereof. The nodes remained in the previously dissected subcutaneous space. The synthesis of the skin was performed with continuous transdermal suture not anchored with polyglactin 4.0 (NOVOSYN®, B.Braun Surgical S.A., Barcelona, Spain).

The analgesia in the immediate postoperative period was performed with paracetamol drops at a concentration of 200 mg/mL diluted in 100 mL of water and offered ad libitum. In post-operative, daily observations of the wound and of the general conditions of the animal were performed to identify and record possible post-operative complications (dehiscence, evisceration, seroma, hematoma, surgical site infection, and death).

**Figure 3:**
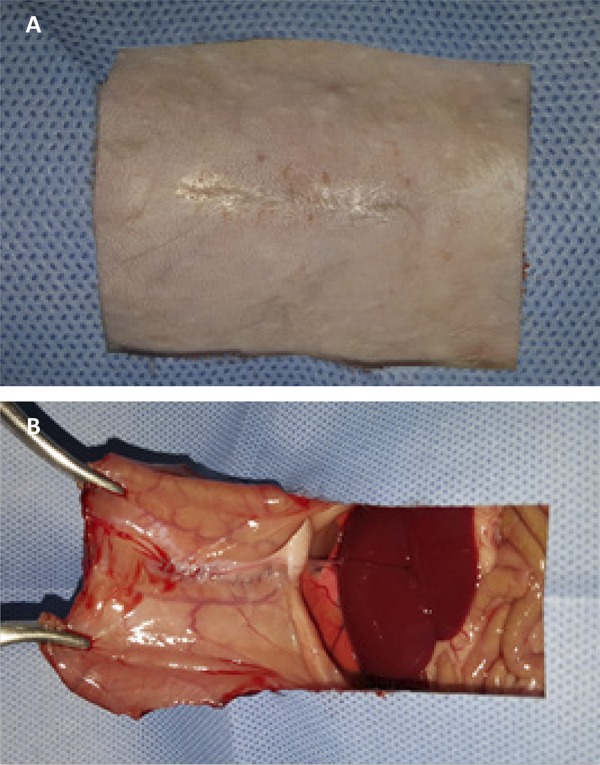
Reopening of abdominal wall in “U-shaped” (A and B).

**Figure 4:**
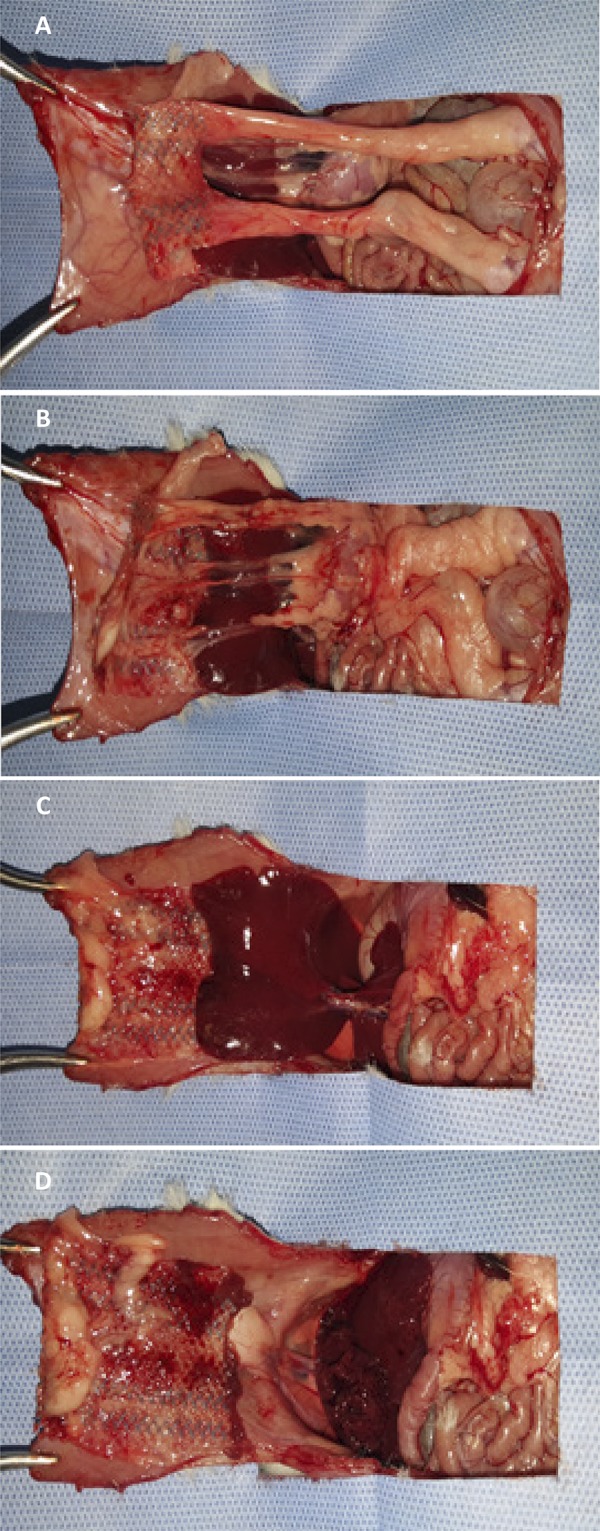
Adhesions with bilateral epididymal fat (A), omentum (B), liver (C), and lysis of adhesions between the mesh and the abdominal viscera (D).

After 21 days, the rats were killed with a mixture of 2% xylazine hydrochloride at the dose of 40 mg/kg and 10% ketamine hydrochloride at the dose of 400 mg/kg intramuscularly. Death was characterized by respiratory arrest and complete absence of reflexes. A U-shaped incision was made involving all anatomical planes of the anterior abdominal wall, bordering the lateral borders of the abdominal wall (lateral borders) and the groin (lower border). The flap remained attached only to the costochondral border (Fig. 3). An analysis of the possible adhesions between the abdominal visceral structures and the visceral surface of the implanted meshes was carried out, in addition to an evaluation of the degree of adhesions according to Lamber et al.^16^(Table 1), as well as the determination of which organs were involved in this process and which sectors of the mesh were compromised, according to Figure 4. Each mesh was evaluated with a grid of nine quadrangular sectors: eight peripheral and one central sector.

**Table 1:** Classification of the degree of adhesions between the abdominal viscera and the surface of the visceral face of intraperitoneally implanted meshes16.

Type of Adhesions	Intensity of adhesions	Definition
0	None	Absence of adhesions
1	Little	Fine adhesions for easy release
2	Moderate	Adhesions requiring a blunt dissection to be released
3	Intense	Firm adhesions, which require a higher force to release them, producing partial or total injury of the viscera involved.

In the analytical statistical evaluation, the Fisher’s exact test was used for the qualitative variables. The test was bilateral with a significance level of 5% (p value<0.05). For analysis of quantitative variables, we used the ANOVA analysis of variance for parametric data and the Kruskal-Wallis test for non-parametric data. The test was bilateral with a significance level of 5%. The analysis of variance was performed by F test, and the normality test by Shapiro-Wilk test. The results of analytical statistics were presented as tables and box-plot graph. The software BioEstat®, version 5.3 (AnalystSoft), was used for statistical analysis.

## Results

There were no statistically significant differences in initial (D1) and final (D21) weight of the animals among the three groups according to the ANOVA test (p value=0.652 for the initial weight, and p value=0.736 for the final weight). However, in all groups, there was a significant increase in the weight of the animals at the end of the study (D21) compared to the weight of the animals at the beginning (D1), according to the ANOVA test (p value<0.0001). The mean initial weight of the animals was 307 ± 33 g, and the mean final weight of the animals was 349 ± 35 g.

**Figure 5:**
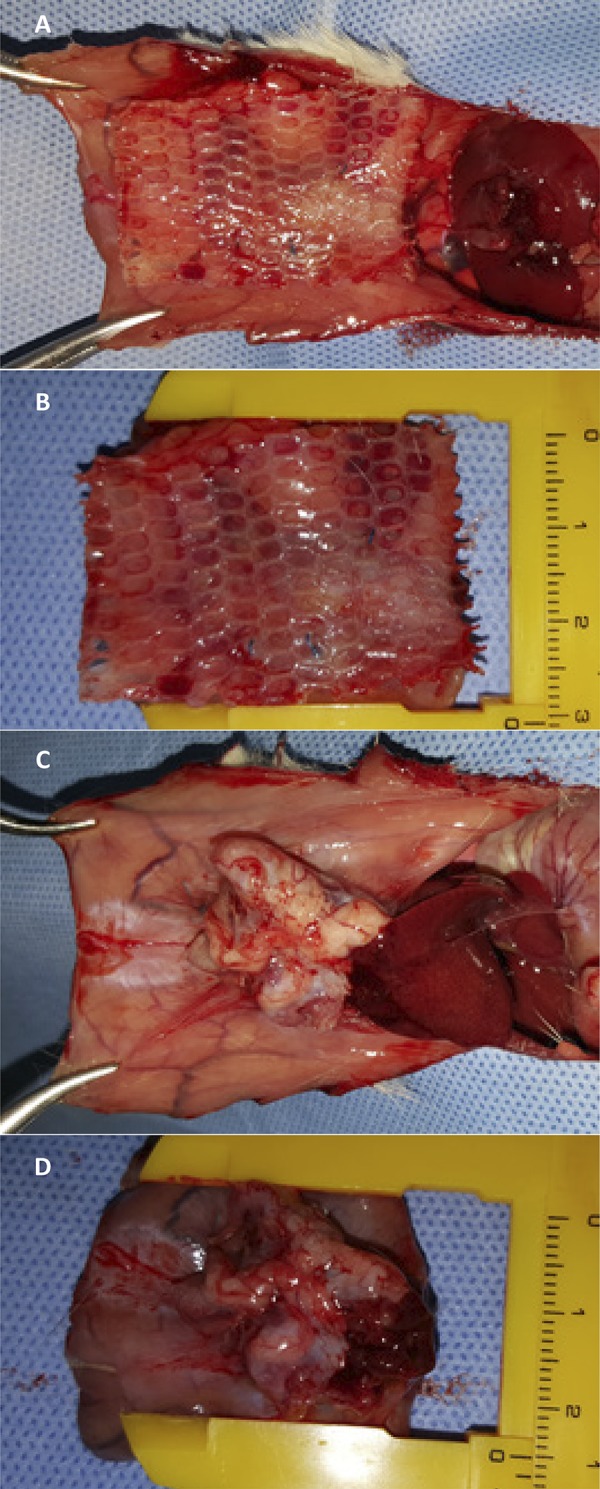
Macroscopic aspects of the polyester/porcine collagen mesh after 21 days of intraperitoneal implantation in rats (A and B), and meshoma of the mesh after partial dehiscence of the operative wound (C and D).

In all groups, there were postoperative complications, with emphasis on partial dehiscence of the operative wound, which was diagnosed between postoperative D7 and D9. However, there were no significant differences between groups (Table 2). At the end of the study, three animals had meshoma, one in the polypropylene/polyglecaprone group and two in the polyester/porcine collagen group (Fig. 5). There were no deaths in any of the study groups.

**Table 2:** Postoperative complications for intraperitoneal implant of polypropylene mesh *versus* polypropylene with polyglecaprone *versus* polyester with porcine collagen in rats for correction of abdominal wall defect.

Complications Post-operative	Polypropylene (10 animals)	Polypropylene Polyglecaprone (10 animals)	Polyester Collagen (10 animals)	p-value
Animals with complications	4 (40%)	8 (80%)	4 (40%)	0.147
Operative wound dehiscence	3 (30%)	8 (80%)	4 (40%)	0.122
Ulcer in left thigh	0	3 (30%)	0	0.089
Difficulty of flexion of the thigh	1 (10%)	0	0	>0.999
Meshoma	0	1 (10%)	2 (20%)	0.754
Death	0	0	0	>0.999

*Fisher’s exact test (Freeman-Halton) - α = 5% (bilateral)

**Figure 6:**
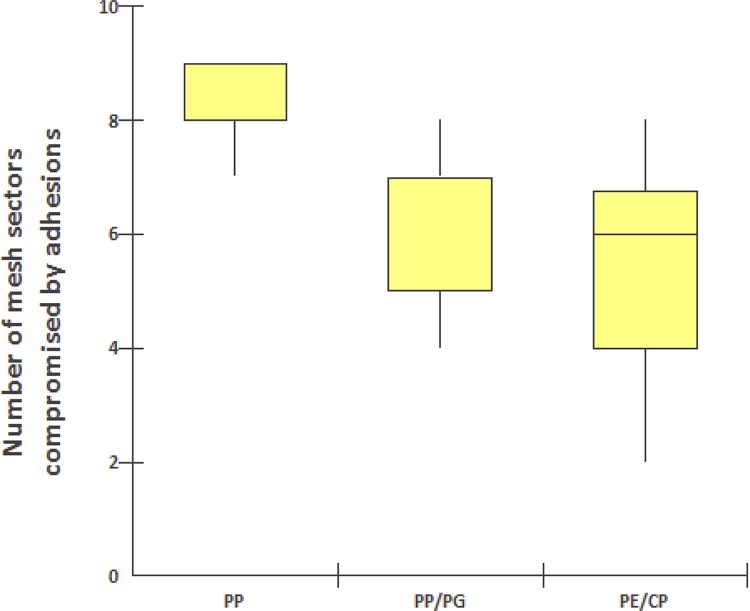
- Number of mesh sectors compromised by adhesions between abdominal anatomical structures and the visceral face of polypropylene (PP), polypropylene/ polyglecaprone (PP/PG), and polyester/porcine collagen (PE/CP) implanted intraperitoneally in rats.

In relation to the intra-abdominal structures compromised by adhesions with implanted meshes, the epididymal fat, omentum and liver were the most involved in all groups. However, there were no significant differences among the three groups, except for the lower number of adhesions with the liver in the polypropylene/polyglecaprone group (Table 3). The polypropylene mesh group presented a statistically significant greater impairment of the surface of the mesh involved with the visceral adhesions (Fig. 6) from both peripheral sectors (p value=0.005) and from the central sector (p value=0.003) in relation to the polypropylene/polyglecaprone and polyester/porcine collagen groups, which did not present significant differences among themselves (Table 4).

**Table 3:** Anatomic structures compromised in the formation of adherences with polypropylene meshes *versus* polypropylene with polyglecaprone *versus* polyester with porcine collagen implanted intraperitoneally in rats for correction of abdominal wall defect.

Compromised anatomical structures	Polypropylene (10 animals)	Polypropylene Polyglecaprone (10 animals)	Polyester Collagen (10 animals)	p-value
Epididymal fat	10 (100%)	9 (90%)	6 (60%)	0.094
Omentum	10 (100%)	10 (100%)	10 (100%)	-
Liver	10 (100%)	6 (60%)	10 (100%)	0.023
Small intestine	0	0	0	-
Cecum	1 (10%)	0	0	>0.999
Bladder	1 (10%)	0	0	>0.999

*Fisher’s exact test (Freeman-Halton) - α = 5% (bilateral)

Median: PP = 8, PP/PG = 5 and PE/CP = 6. Kruskal-Wallis: PP x PP/PG = 0.0030 - PP x PE/CS = 0.0014 – PP/PG x PE/CP = 0.8192

**Table 4:** Location of adhesions in peripheral and central sectors between abdominal anatomical structures and the visceral face of meshes of polypropylene *versus* polypropylene with polyglecaprone *versus* polyester with porcine collagen implanted intraperitoneally in rats for correction of abdominal wall defect.

Location of adhesions	Polypropylene (10 animals)	Polypropylene Polyglecaprone (10 animals)	Polyester Collagen (10 animals)	p-value
Peripheral	72 (88%)	55 (95%)	52 (96%)	*0.005
Central	10 (12%)	3 (5%)	3 (4%)	**0.003
Total	82 (100%)	58 (100%)	55 (100%)	*0.002

*Kruskal-Wallis test - α = 5% (bilateral)

**Fisher’s exact test - α = 5%(bilateral)

P-value = 0.005 - Polypropylene *versus* Polypropylene/Polyglecaprone *versus* Polyester/Porcine Collagen in relation to adhesions in peripheral sectors.

P-value = 0.003 - Polypropylene *versus* Polypropylene/Polyglecaprone *versus* Polyester/Porcine Collagen in relation to adhesions in central sectors.

P-value = 0.002 - Polypropylene *versus* Polypropylene/Polyglecaprone *versus* Polyester/Porcine Collagen in relation to total adhesions in all peripheral sectors.

**Figure 7:**
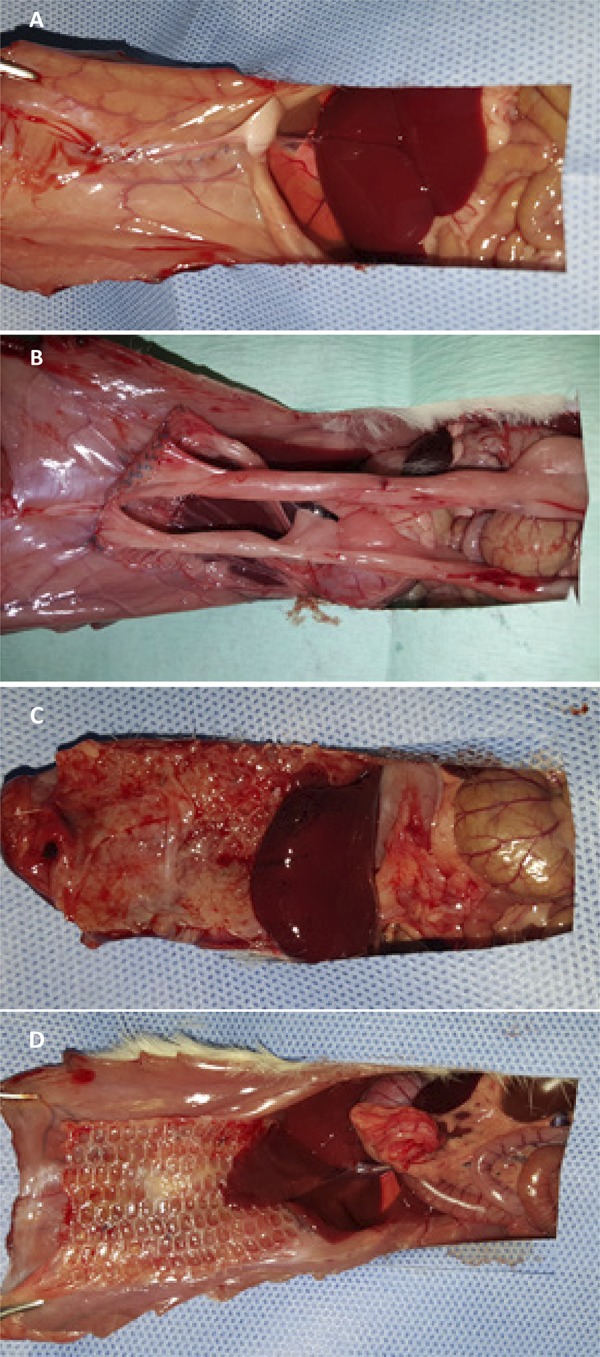
Aspect of the peritoneal cavity and of the meshes after 21 days of the intraperitoneal implant in rats: (A) without the use of mesh, (B) polypropylene mesh, (C) polypropylene/polyglecaprone mesh and (D) polyester/porcine collagen mesh.

In relation to the intensity of the adhesions between the intra-abdominal structures and the implanted mesh, the polypropylene mesh group presented a higher degree of statistically significant intense adhesions in relation to the polypropylene/polyglecaprone and polyester/porcine collagen groups (p value<0.001) (Table 5). On the other hand, the polyester/porcine collagen composite group also presented a higher degree of intense adhesions with statistical significance in relation to the polypropylene/polyglecaprone group (p value=0.035), although there was no significant difference in relation to the degree of adherence between both groups (p value=0.289) (Fig. 7).

**Table 5:** Degree of adhesions in abdominal anatomical structures and the visceral surface of meshes of polypropylene *versus* polypropylene with polyglecaprone *versus* polyester with porcine collagen implanted intraperitoneally in rats for correction of abdominal wall defect.

Degree of adhesions	Polypropylene (10 animals)	Polypropylene Poliglecaprone (10 animals)	Polyester Collagen (10 animals)	p-value
Little	1 (1%)	1 (2%)	3 (5%)	0.302
Moderate	4 (5%)	33 (57%)	19 (35%)	0.289
Intense	77 (94%)	24 (41%)	33 (60%)	0.035
Total	82 (100%)	58 (100%)	55 (100%)	< 0.001

*Fisher’s exact test - α = 5%(bilateral)

P-value < 0.001 - Polypropylene/Polyglecaprone *versus* Polyester/Porcine Collagen in relation to intense adhesions.

P-value = 0.289 - Polypropylene/Polyglecaprone *versus* Polyester/Porcine Collagen in relation to moderate adhesions.

P-value = 0.035 - Polypropylene/Polyglecaprone *versus* Polyester/Porcine Collagen in relation to intense adhesions.

## Discussion

All meshes showed some degree of adhesion in all experiments. However, the tissue separating meshes composed of polypropylene/polyglecaprone and polyester/porcine collagen presented a statistically significant lower impairment (p value=0.002) of their visceral surface by adhesions with intra-abdominal organs and structures compared to the polypropylene mesh. Ditzel *et al*.^18^, in a study with rats with implants of five different types of meshes, among which polypropylene and polyester collagen meshes, concluded that the polyester composite mesh with collagen showed a significantly small surface of adherence compared to the polypropylene mesh. Lamber *et al*.^16^ developed a similar study with an intraperitoneal mesh implant of polypropylene and polyester with collagen in rats, and concluded that the polypropylene mesh showed a significantly greater adhesion compromise than the polyester mesh with collagen. Similar results were found by Garcia *et al*.^19^ in an experimental study with rabbits with polypropylene mesh versus polypropylene with bovine collagen, where animals with only a polypropylene mesh presented a greater surface area of adhesion compromise with intra-abdominal viscera compared to the double-sided mesh. However, the result of the present study differs from the study by Biondo-Simões *et al*.^20^, in which there was no significant difference in the surface compromised by adherences between the meshes after intraperitoneal implantation of the polypropylene and polyester meshes with collagen in rats.

The use of polypropylene-only meshes in direct contact with the viscera and intra-abdominal structures resulted in extensive impairment of visceral adhesions. The use of polypropylene-only meshes for the repair of defects in the abdominal wall and in intimate contact with the intra-abdominal viscera, therefore without any separating films, is associated with the appearance of dense and firm adhesions^21^. Paulo *et al*.^22^, in a study involving rats and polypropylene mesh implants versus polypropylene with poly-2-hydroxyethyl methacrylate (polyHEMA) hydrogel for abdominal wall defect correction, concluded that the composite mesh of polypropylene alone determined the appearance of adhesions throughout the surface of the mesh. In contrast, the mesh of polypropylene with hydrogel presented low numbers of adhesions with intra-abdominal viscera.

On the other hand, there was no significant difference in the compromised surface among the tissue-separating meshes used in the study. Thus, although the double-sided meshes are designed to avoid the appearance of adhesions with the intra-abdominal viscera at their visceral surface, when they are positioned intraperitoneally^9^ their use has been shown to be incapable of preventing the formation of adhesions in their fullness. Schulz *et al*.^23^, in a study with rabbits, found that there was no significant difference in adhesion formation between bovine collagen and bovine collagen-elastin polyester meshes when implanted in direct contact with the intra-abdominal viscera.

In an experimental study comparing fourteen different types of meshes commonly used for the repair of abdominal wall hernia defects involving synthetic and non-barrier (tissue separating) meshes and biological artificial prostheses, Gaertner *et al*.^14^ concluded that all meshes determined the appearance of visceral adhesions. Non-stick barrier-free synthetic fabrics have a more extensive adherence compromise. Biological meshes had a significantly lower area of impairment. In turn, the tissue-separating synthetic meshes had an area of adhesion comparable to biological ones. In an experimental study, Lamber *et al*.^16^, comparing a pollen mesh covered with porcine collagen and one with polypropylene, reached a similar conclusion. Both meshes determined the appearance of adhesions, although the tissue-separating mesh presented a much lower compromising surface than the non-stick barrier polypropylene mesh.

In a review on the choice of tissue material for intraperitoneal disposition in the surgical treatment of abdominal wall hernia defects, Araújo *et al*.^13^ concluded that there is no consensus as to which is the best material for the composition of the prosthesis that should be placed inside the abdominal cavity in direct contact with viscera. However, the authors suggested that the prosthesis should be made preferably of synthetic material, reticular and macro-porous, in order to stay in contact with the musculature, and of another laminar material and micro-porous, in order to stay in contact with the viscera.

The intraperitoneal postoperative adhesions are the anatomical expression of a pathological healing process, which can arise after any injury that compromises the parietal or visceral peritoneum, especially as a result of the surgical manipulation of the cavity and abdominal organs. Its etiopathogeny is intimately related to changes in fibrinolysis^24^. This process involves inflammatory cells such as neutrophils, monocytes and macrophages, as well as the release of inflammatory mediators and cytokines such as tumor necrosis factor alpha, fibrin synthesis, fibroblast migration, and collagen synthesis. The increase in inhibitors of plasminogen activating factor determines the impairment of plasmin synthesis, which reduces the degradation of fibrin formed during the initial inflammatory phase of the tissue repair process^24^. The consequent reduction in fibrinolysis contributes to the organization of the fibrin matrix, which determines the appearance of adhesion points between two opposing injured peritoneal surfaces. The migration of fibroblasts contributes to the synthesis of several types of collagen and adhesion maturation, which is also marked by neovascularization and the appearance of new nerve branches and mesothelialization. The presence of suture threads and prosthetic material, such as reticulated meshes used for the repair of abdominal wall defects, are considered as adjuncts contributing to the formation of intraperitoneal adhesions, which, in direct contact with the injured peritoneal surface, contribute to the appearance of adhesions^24-25^.

The involvement of peripheral sectors of the mesh by adhesions with the viscera and intra-abdominal structures was statistically significant (p value<0.005) for the mesh composed exclusively of polypropylene compared to the tissue-separating meshes (polypropylene/polyglecaprone and polyester/porcine collagen), which did not show any differences between them. However, the number of peripheral sectors involved in double-sided meshes was high. In fact, most adhesions observed containing these tissue-separating meshes involved the peripheral sectors of the implanted prosthesis. However, they did not compromise the central sector. The preparation of the mesh fragment to be implanted in the model of the experiment allowed that its edge did not have the protection of the non-stick layer, exposing the reticulated layer of the mesh, which favored the appearance of adhesions in these peripheral sectors. Therefore, sectioning the tissue separation mesh to be implanted makes it vulnerable to the appearance of adhesions and compromises its effectiveness.

The involvement of central sectors of the mesh by adhesions with the viscera and intra-abdominal structures was statistically significant (p value=0.003) for the mesh composed exclusively of polypropylene compared to the tissue-separating meshes (polypropylene/polyglecaprone and polyester/porcine collagen), which did not show any differences between them. It was evident that this central sector is not compromised in double-sided meshes, where the visceral layer of the mesh is intact and was not compromised by the prosthesis section during the experiment. In fact, most meshes separating implanted tissues did not show adhesions in the central sector of the mesh. This behavior was credited to the presence of the tissue-separating mesh on the visceral surface of the double-sided mesh, which effectively prevented adhesions from appearing. Although the use of tissue separating meshes reduces the probability of adhesions, this was not able to neutralize the appearance of adhesions completely.

The polypropylene mesh not only determined a more extensive impairment of its visceral surface by adhesions with the viscera and intra-abdominal structures, but also resulted in the appearance of a higher number of intense adhesions in comparison with the tissue-separating meshes (polypropylene/polyglecaprone and polyester/collagen), with a significant statistical significance (p value<0.001). This outcome, although expected, ratifies the risk associated with the use of polypropylene meshes in direct contact with the intra-abdominal viscera and the appearance of extensive and firm adhesions between the visceral surface of the mesh and the abdominal organs. Garcia *et al*.^19^ concluded that the polypropylene mesh, although effective in treating abdominal wall defects, causes an intense inflammatory and foreign body reaction. The appearance of dense and firm adhesions with the intra-abdominal viscera is attributed to this bioincorporation process of the intraperitoneally implanted mesh. Ricciardi *et al*.^26^ performing an experimental model in rats, confirmed that a polypropylene mesh implant in direct contact with the intra-abdominal structures is able to generate more intense adhesions. However, when wrapped in an autologous fibrous tissue, it is able to reduce the degree of adhesions. Therefore, it is not recommended to use synthetic meshes made exclusively of polypropylene for the repair of defects of the abdominal wall in which the prosthesis will be placed intraperitoneally and in immediate contact with the intra-abdominal viscera.

Polypropylene/polyglecaprone and polyester/porcine collagen composite meshes presented a similar behavior regarding the appearance of moderate adhesions between the visceral surface of the mesh and the intra-abdominal organs and structures, therefore without a statistical significance (p-value=0.289). However, the polyester/collagen mesh presented a behavior marked by more intense adhesions compared to the polypropylene/polyglecaprone composite mesh, which was associated with a statistical significance (p value=0.035). Both tissue-separating meshes are suggested for use in intraperitoneal techniques for the repair of abdominal wall hernia defects, but the polypropylene/polyglecaprone mesh was more effective in reducing the adhesion intensity in the present study.

In a review of the composition, characteristics and efficiency involving eight different types of tissue separating meshes used for the repair of ventral hernia, Deeken *et al*.^17^ concluded that the differences observed among the various anti-dense barrier meshes are commonly attributed to the chemical composition or conditions necessary for reabsorption and metabolism of its components. It is a complex process and several factors should be taken into account. Usually, the components of these barriers determine a wide variety of inflammatory responses and fibroplasia, resulting in a diverse rate of adhesions between the mesh and intra-abdominal viscera^27^.

The performance of synthetic meshes available for the repair of abdominal wall hernias is determined by factors related to the polymer used to make the mesh, to the structural conformation of the mesh, which is influenced by the type of polymer fiber composing it, textile characteristics and porosity of the mesh, prosthesis and the interaction between the mesh and the tissue^9^. However, this mechanistic view that the biomechanical properties of the meshes would guarantee the success of hernioplasty is being rethought. The biological response to the presence of the mesh had a key role in the outcome of the treatment performed here. The understanding that the mesh determines structural changes in the tissues with which it comes into contact, as well as the counterpart of the impact of the biological process of incorporation of the mesh, marked by the balance of inflammation response and fibroplasia, decisively influence the result of the repair with synthetic meshes of abdominal wall defects. It refers to the need to create a mesh-tissue integration index as a new reference for evaluating the performance of a synthetic mesh^28^.

An appropriate biological response favors a healthy biointegration of the tissue capable of determining a greater flexibility of the prosthesis and respect for anisotropy of the abdominal wall, less probability of hernia recurrence, and chronic pain. On the other hand, an excessive integration response results in a greater retraction and hardening of the mesh, as well as a greater likelihood of hernia recurrence, chronic pain, adhesions, and fistulas. Therefore, the success of a synthetic mesh implant depends essentially on the dual-handed pathway established between the mesh and the tissue^9-28^.

There is no consensus about the best score to be used to assess the intensity of adhesions formed between the mesh and the organs and intra-abdominal structures in experimental animal studies^20^. There is a subjectivity regarding the stratification used in the present study to analyze the intensity of the degree of adhesions, especially the intensity of the force used to remove them. The very complexity of the factors involved with the inflammatory response and fibroplasia subsequent to the mesh implant is capable of modulating the results on the formation and intensity of intraperitoneal adhesions^24^. This scenario limits the comparisons between different researches and results found in the medical literature.

Although there were no statistically significant differences among the three groups in relation to postoperative complications, it is important to note that the presence of partial dehiscence of the operative could compromise the bioincorporation process of the synthetic mesh, favoring the contamination and consequent infection of the surgical site and the implanted mesh. In the present study, the occurrence of meshoma was observed in three animals that had a partial dehiscence of operative wound and closure by second intention without the use of dressings or antibiotics. The infection of the operated site determines a prolonged inflammatory response and directly compromises the fibroplasia phase and collagen deposition, favoring the appearance of more intense adhesions, chronic infection of the mesh, retraction of the prosthesis, and appearance of meshoma^28^. In a clinical scenario, this outcome represents an important risk factor for complications such as visceral adhesions, chronic pain, intestinal obstruction, fistulization, hernia recurrence, and reoperations^14-15^. Therefore, the presence of complications related to the operative wound, such as dehiscence, favors the contamination and infection of the prosthesis, which represents an important risk factor for the failure of the synthetic mesh implant in the treatment of abdominal wall defects^29^.

The use of preclinical animal models to assess the biocompatibility, efficiency, and specific characteristics of meshes with innovative concepts is an indispensable part of the research on abdominal wall hernias and prostheses to be used for a proper hernia repair. Important properties of meshes related to mesh-tissue interaction, such as inflammatory response, fibroplasia, bioincorporation, prosthesis retraction, and adhesion formation can only be evaluated in experimental animal studies. Therefore, it is impractical in humans for bioethical reasons^30^. However, there is a great variety in experimental models in animals currently used, such as different types of animals and prostheses, size and shape of the defect created in the abdominal wall, positioning of the mesh in relation to the muscle-aponeurotic plane, fixation of the mesh, size of the mesh used, and intended objectives^14-30^. Such heterogeneity of methods implies limitations and flaws to comparisons between the experimental studies currently used for abdominal wall hernias and meshes^30^.

Extrapolating the findings of an animal model (mice) to a practical application in clinics is somewhat difficult. As in most experimental models involving meshes, we used the creation of the defect in the musculoaponeurotic plane, followed by the immediate implantation of the mesh to repair the defect created in the abdominal wall. However, in a clinical scenario using meshes for the repair of abdominal wall hernias in patients, the prosthesis is usually implanted after the maturation of the defect that gave rise to the hernia. Therefore, the results between the animal experimental scenario and the clinical scenario could be different even if the same mesh and implant were used in the same way^30^.

## Conclusions

The intraperitoneal implantation exclusively of polypropylene meshes to correct defects of the abdominal wall causes the appearance of extensive and firm adhesions with the intra-abdominal structures. The use of polypropylene/polyglecaprone or polyester/porcine collagen tissue-separating meshes reduces the number and degree of adhesions formed.

## Footnotes

1 Research performed at the Experimental Surgery Research Laboratory, Hospital Universitário, Universidade Federal do Maranhão (HU-UFMA), Sao Luis-MA, Brazil. Part of PhD degree thesis, Postgraduate Program in Health Sciences, Faculty of Medical Sciences, Universidade do Estado do Rio de Janeiro (UERJ). Tutors: Prof. Marcos Bettini Pitombo, and Prof. Orlando Jorge Martins Torres.
